# Athletic trainers’ viewpoints of patient-centered care: Preliminary findings

**DOI:** 10.1371/journal.pone.0274577

**Published:** 2022-09-14

**Authors:** Carly J. Wilson, Lindsey E. Eberman, Ansley S. Redinger, Elizabeth R. Neil, Zachary K. Winkelmann

**Affiliations:** 1 Department of Exercise Science, University of South Carolina, Columbia, South Carolina, United States of America; 2 Department of Applied Medicine and Rehabilitation, Indiana State University, Terre Haute, Indiana, United States of America; 3 St. Luke’s Health System, Boise, Idaho, United States of America; 4 College of Public Health, Temple University, Philadelphia, Pennsylvania, United States of America; Rosalind Franklin University of Medicine and Science Doctor William M Scholl College of Podiatric Medicine, UNITED STATES

## Abstract

The core competency of patient-centered care (PCC) states that for positive patient outcomes, the provider must respect the patient’s views and recognize their experiences. The Athletic Training Strategic Alliance Research Agenda Task Force identified a profession-wide belief that examining the extent to which athletic trainers (ATs) provide PCC in their clinical practice would benefit the profession. To first address this line of inquiry, we must study the subjectivity of how ATs view PCC. This study used Q methodology which is a research design that collects data from participants from a quantitative and qualitative perspective. A total of 115 (males = 62, females = 53, age = 37±10 y, experience = 13±10 y) ATs dispersed between 11 job settings volunteered for this study. Participants were asked to pre-sort (agree, disagree, neutral) 36 validated statements representing the 8 dimensions of PCC, then completed a Q-sort where they dragged-and-dropped the pre-sorted statements based on perceived importance in providing PCC. The Q-sorts were analyzed using QMethod software. A principal component analysis was used to identify statement rankings and factors. Factors were determined by an Eigenvalue > 1 and analyzed using a scree plot. The 6 highest selected statements per factor were assessed to create the distinguishing viewpoints. Two distinguishing viewpoints emerged from the factor analysis of the Q-sorts: 1) the interpersonal connection that valued teamwork, open communication, and respectful care with varied populations; 2) the holistic gatekeeper that valued personal promotion for activities of daily living, self-care, and quality of life. Overall, ATs value patient’s preferences and respect. However, a lack of importance was identified for incorporating the disablement model which is a core competency and adopted framework by the athletic training profession.

## Introduction

The Institute for Healthcare Improvement created the Triple Aim for Healthcare which is a set of linked missions with the intent to raise the standards of the United States healthcare system. The long-term goal of the Triple Aim for Healthcare is the provision of excellent care will ultimately enhance the patient experience [1]. These key areas include reducing the per capita cost, improving the health of the population, and providing better care that enhances the patient experience [1]. As detailed in previous literature, patient satisfaction is dictated by the value that the provider places on the patient themselves [2]. This concept is referred to as patient-centered care (PCC) whereby the provider is responsive to and respectful of the patient’s values and needs in their clinical care [3–5]. However, most providers are guided by the “Golden Rule” in that we treat others as we wish to be treated [6]. As defined by the National Academy of Medicine (formerly Institute of Medicine), PCC is care that is respectful of and responsive to each patient’s preferences, desires, and beliefs through a shared-decision making model [7, 8]. The medical literature has specifically identified eight components of PCC which include 1) respect for patient preferences, values, and expressed needs, 2) information, education, and communication, 3) coordination and integration of care and services, 4) emotional support, 5) physical comfort, 6) involvement of family and friends, 7) continuity and transition, and 8) access to care and services [7, 9]. Some of the benefits from approaching care through a patient-centered philosophy includes improved health outcomes and higher satisfaction [10].

Patient-centered care, while regarded as a key component of high-quality medical care, lacks operational consistency and valid measurements when compared across numerous medical professions [11]. The skill of delivering PCC has often been categorized as a structure of healthcare itself versus specific to a provider [9, 12]. Although the principles of PCC, such as access to care, coordination and integration of care, and identification of a social support system, are true across healthcare professions, the provider may be highlighted and/or diminished depending on their job setting or specialty area [13]. It is believed that the first interaction with a provider can often dictate the patient’s future intentions to seek out care, leading to long-term negative health consequences if the initial visit communication was disappointing to the patient [14]. Negative interactions with healthcare providers are guided by clinicians speaking in medical jargon, not giving the patient a voice, patient education that is not reflective of one’s literacy level, and implicit biases related to the injury or illness. These factors often influence how much effort is placed into delivering care that is centered on the person, rather than the disease. Previous literature investigated the relative importance of the 8 principles of PCC from the perspectives of healthcare professionals in geriatric and surgical intensive care hospital units [7]. Through the analysis, the researchers concluded that viewpoints on important elements for PCC differed more among medical professionals between the two departments, but overall, patient preferences, information and education, and coordination of care dimensions were considered to be the most important PCC principles [7]. However, due to their lack of generalizability, the research team specifically called for supplemental research with other healthcare professions to see if the delivery and opinions of PCC varied across the medical community [7].

Athletic training is a unique profession with direct access to most patient populations that influences the patient-provider relationship relative to trust, communication, and coordination of care. Therefore, the principles of PCC, while still fundamentally identified the same way, may be expressed in terms of importance by the practicing athletic trainer (AT). Currently, most ATs (like other healthcare professionals) practice from a clinician-driven mindset that is focused on identifying the diagnosis and prescribing interventions for healing [12]. Moreover, there has been a long-standing concern within athletic training relative to independent medical care free of influence or bias [15]. The concern is rooted in the athletics delivery model where coaches and administrators often dictate the provision of care [15]. Recently, 18% of collegiate athletes have reported that an AT allowed or made them participate when they should have been medically disqualified from participation noting the continued issue relative to healthcare delivery in sports medicine [16]. Within athletic training, PCC directly explores how and why an injured or ill individual may heal while others with chronic concerns are slow to get better [10, 17]. This leads us to believe that a PCC approach would help us to push aside the “golden rule” of treating others how you would want to be treated and begin providing care that is responsive to the patient’s specific needs [6]. While the patient, as a consumer, is the ultimate voice for patient satisfaction and effectiveness of communication, we must first identify how ATs place importance on the principles of PCC and to examine the context of their viewpoints. For the purpose of this study, *viewpoints* are considered the distinct perspectives held by the athletic trainers related to PCC. Therefore, the objective of this research study was to determine how athletic trainers place importance on the principles of PCC and to examine the context of their viewpoints. We predicted that job setting would have an impact on athletic trainers’ viewpoints of PCC.

## Methods

### Study design

The theoretical basis for this project was based on viewpoints and opinions using the Q methodology through a cross-sectional study design. Typically, viewpoints and opinions are studied using qualitative interviews, whereas Q methodology is the combination of qualitative and quantitative research tactics that allows subjective data from individuals to be assessed and correlated through factor analysis [18]. Q methodology allows for the participant to share their perspective from a positive, negative, or neutral stance with an emphasis on ranking some opinions as more important than others. This unique method was created in order to identify different qualitative patterns of thought, particularly the “how” and “why” thought processes of involved subjects [19]. Utilization of Q methodology techniques and publication of its results will ultimately show the benefits of collecting both the “how” and the “why” people think the way they do. This investigation was deemed exempt research by the University of South Carolina Institutional Review Board (Protocol # 00096882). Participants provided written consent electronically in the survey before proceeding to the questions in the instrument.

### Instrument

The instrument for this study followed the Q methodology, which is a direct response to the issues related to Likert-scale research by which comparisons between and within groups is often limited to minute changes [20]. Q methodology allowed for the research team to explore the participant’s viewpoints while not solely focusing on agree-disagree scales, but simultaneously exploring the research question from a quantitative, ranked perspective using an opinion statement list [21].

For the purposes of this study, the authors explored the PCC literature to identify a PCC opinion statement list. In doing so, the authors identified a 35-item statement list following the eight principles of PCC that was validated and studied in a population of healthcare providers working in the geriatrics and surgical intensive care units of a large teaching hospital [7]. The previous tool underwent pilot testing with hospital and outpatient clinic providers and was used in previous Q methodology research [7]. The nature of Q methodology also did not warrant a need for validity and reliability because the statement list consists of evidence-based phrases derived from literature that are written as opinion statements. As the 35-item statement list was written in terms of physicians and hospital experience, the research team adapted the list to the profession of athletic training by making small verbiage changes from “healthcare professionals” to “athletic trainer”. The adaptation of the statement list was performed by two members of the research team (AAA, BBB) and underwent face validation by the three additional members of the research team (CCC, DDD, EEE), which was ultimately used to build the final PCC item statement list. During the face validation process, one statement was added *(“Athletic trainers integrate the International Classification of Functioning*, *Disability*, *and Health (ICF) model as a framework for delivery of patient care*.*”)* based off the educational standards in athletic training relative to patient-centered care. The final PCC statement list contained 36 items split into the eight principles including patients’ preferences (7 items), physical comfort (4 items), coordination of care (4 items), emotion support (3 items), access to care (6 items), continuity and transition of care (4 items), information and education (5 items), and support system (3 items). The full list containing the 36 PCC item statements is provided in S1 Table.

### Participants

The sample for this study was chosen for diversity rather than quantity, which is based off previous Q methodology in healthcare studies [7, 19]. Respective to Q methodology, the P set (or the participants) should be a purposeful sample of a specific population. The quantitative number of subjects is not as crucial for this type of study as it is for survey studies because the analysis is not focusing on the numerical value of participants thinking in a certain pattern. Thus, sample sizes tend to be on the smaller end because the results will not be negatively affected by low response rates [22].

The participants in this study were certified ATs in the United States and members of the National Athletic Trainers’ Association (NATA). At the onset of this study, 3680 eligible ATs were contacted and sent the recruitment information via the membership e-mail list; 399 surveys were started. After removing those who were not a certified athletic trainer or did not consent, 285 individuals were then presented the link to access the Q-sort. Out of the 285 responses, 116 participants successfully completed the Q-sort. One participant did not report demographic data and was removed bringing our final sample size to 115 ATs (age = 37 ± 10 y; male = 62, 53.9%, females = 53, 46.1%; means years of experience = 13 ± 10 y, experience range = 1 to 40 y) with 102 of those responses completing the final open-ended response portion of the survey.

### Procedures

Participants were sent an e-mail with an invitation to participate via a web-based survey (Qualtrics, Inc., Provo, UT) in the Spring of 2020. After agreeing to participate, the participant clicked on a link to the Q Method survey and input their unique participant code. A distribution reminder was sent weekly after the initial contact for one month. After written informed consent was obtained, participants were given instructions to fully complete the survey. All data collected from the web-based survey was automatically stored and recorded into a Q methodology software (QMethod Software, Windsor, Ontario) [23].

The survey began by collecting basic demographic information relative to one’s sex, age, job setting within athletic training, and years of experience. Next, the participant was tasked with completing a pre-sort of the 36-item statement list. To perform the pre-sort, each statement was independently provided with scale labels (agree, disagree, neutral). The pre-sort phase allowed participants to express personal importance for PCC principles by clicking one of the scale labels to place that statement into a category.

In the Q-sort phase, the participants were prompted to rank the importance of their 36 selected statements based off the pre-sort results via a drag-and-drop feature into a hierarchy table that went from highest agreement to lowest agreement. The purpose of this section was for the participant to take the statements based off their assigned scale labels from the pre-sort and place them throughout the hierarchy table to describe which of those they thought felt matched their viewpoints and values the most. The final portion of the Q methodology included two follow-up, open-ended questions respective to the participant’s Q-sort about why they selected the top and bottom statements on their hierarchy table.

### Statistical analysis

Data were analyzed using the built-in statistical features of the Q Method (PQMethod 2.11 software). First, we performed a Pearson correlation of the 115 Q-sorts to produce a correlation matrix. Next, a principal component analysis (PCA) was performed that identified 8 factors with each factor meeting the Kaiser-Guttman criterion meaning the factor had an Eigenvalue >1.

Next, the Eigenvalues for the factors extracted from the PCA were plotted on a scree plot. A scree plot requires plotting multiple factor Eigenvalues to determine how many to keep in a factor analysis [21]. From the scree plot, we identified 2 factors that had actual Eigenvalues higher than the 95^th^ percentile denoted by the slope change. The data supported a maximum of 8 factors explaining 53.8% of the variance. The 2-factor scree plot solution explained 28.1% of the variance as the most comprehensible for our data interpretation. Next, we performed a varimax rotation for the 2 factors which produced a factor loading saturation. A follow-up centroid factor analysis was completed to confirm the 2 factors extracted during the principal component extraction.

Relationships were then explored between rankings to indicate similar viewpoints amongst the participants in the study. Statements (st.) ranked with a positive numeral value of 5 were considered “highest agreement”, while statements ranked with a negative numeral value of -5 were deemed “lowest agreement.” As the numeral values moved closer to the center of a Q-sort and neutral agreement (factor ranking = 0), the number of statements able to be assigned under the numeral values increased. The six highest and single lowest selected statements per factor were assessed to create the distinguishing viewpoints. The qualitative responses from the open-ended response items complemented the findings to explain the shared views in more detail. These were used to support the findings rather than coded as themes for additional analyses. Alpha levels were *a priori* at *P* < 0.05.

## Results

### Composite Q-sort

**Fig 1:**
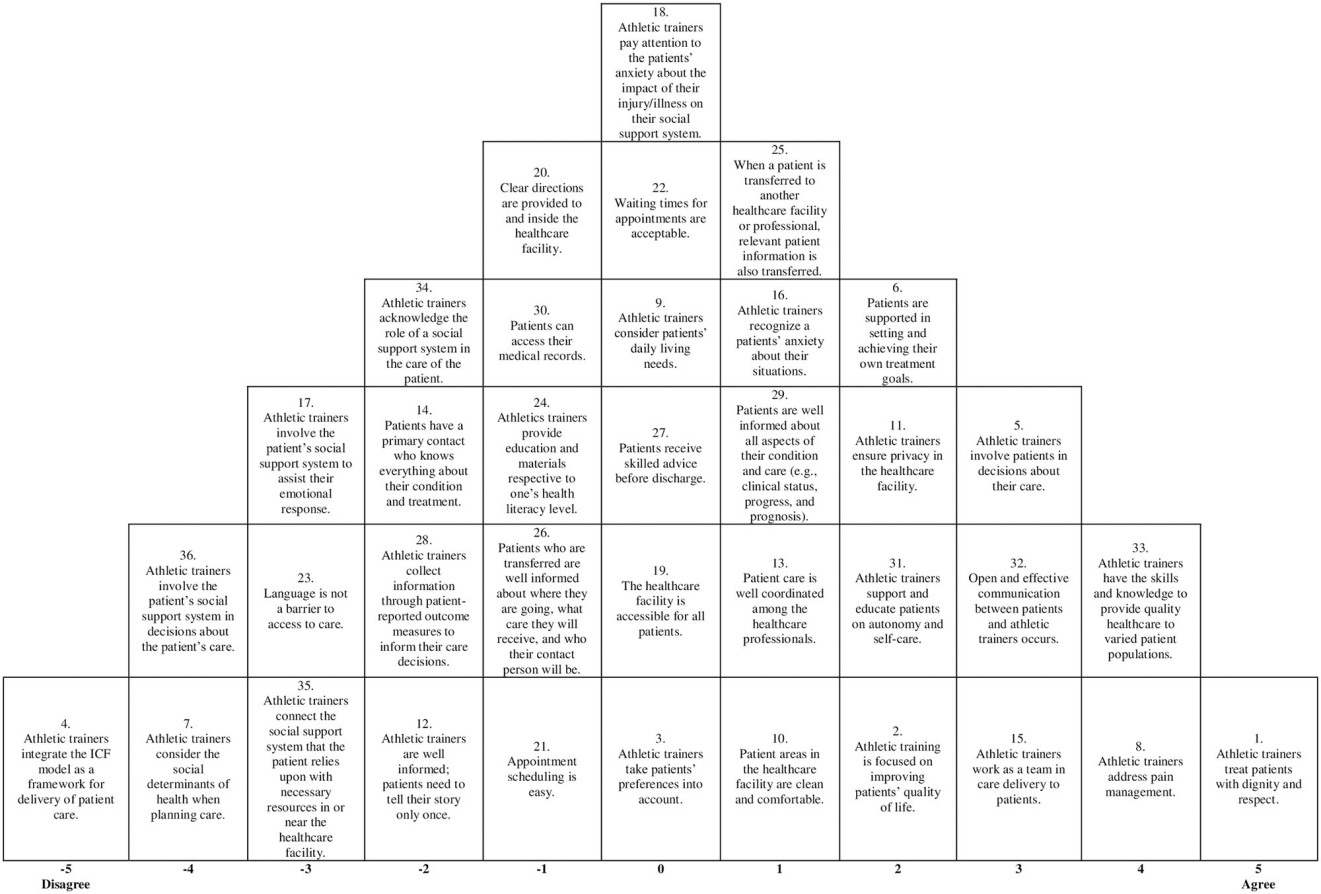
Composite Q-sort 1. The sort further describes the importance of the 36 PCC statements for viewpoint 1.

**Fig 2:**
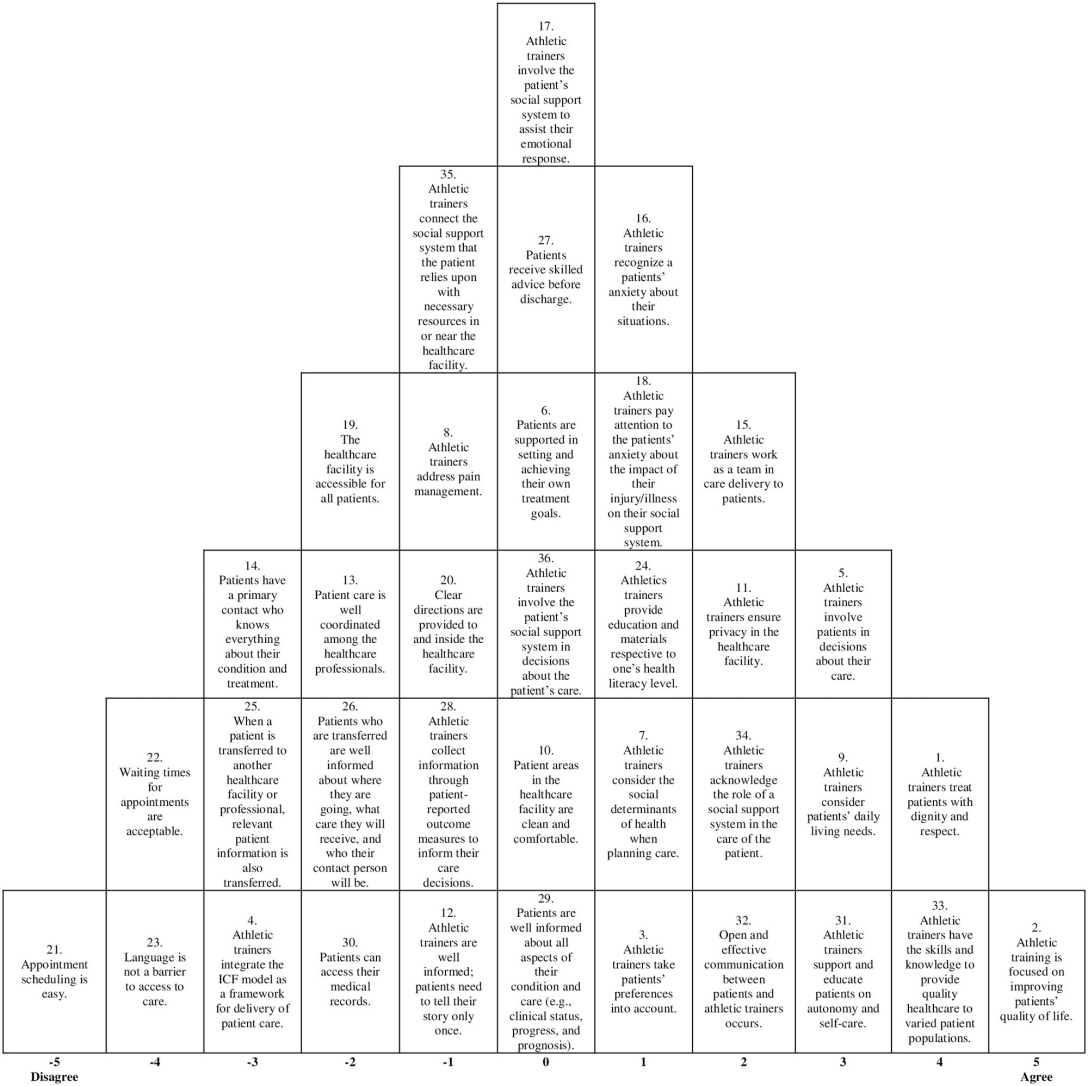
Composite Q-sort for viewpoint 2. The sort further describes the importance of the 36 PCC statements for viewpoint 2.

Two distinguishing viewpoints emerged from the Q-sorts after factor analysis was completed. The factor loading identified each participant’s Q-sort into one of the 2 factor groups identified by majority of common variance. The correlation between the factors was 0.52591 and the covariance produced by the factors was -0.01519 meaning the two factors tend to move in inverse directions. The two viewpoints included 1) the interpersonal connection that valued teamwork, open communication, and respectful care with varied populations; and 2) the holistic gatekeeper that valued personal promotion for activities of daily living, self-care, and quality of life. These two viewpoints were assembled into composite Q-sorts to further represent the rankings of all 36 PCC statements across each factor Q-sort table from highest to lowest. These are displayed in Figs 1 and 2 through a pyramid schematic. The statement “athletic trainers treat patients with dignity and respect” appeared as a top ranked statement in both distinguishing viewpoints, while the lowest ranked statement in viewpoint 1 was “athletic trainers integrate the International Classification of Functioning, Disability, and Health (ICF) model as a framework for delivery of patient care.” Additional open-ended responses received for the highest and lowest statement reasoning section of the survey are compiled each with their respective PCC statement in S2 Table.

### Viewpoint 1: The interpersonal connection

Viewpoint 1 consisted of healthcare professionals who encompassed strong values towards the core PCC principles of including patient preferences into decision-making as well as providing patients with the necessary information and education to succeed in their recovery. Participants within viewpoint 1 reported “athletic trainers treat patients with dignity and respect” (st. 1, factor ranking = 5, Z score = 2.18) as their highest ranked statement on the Q sort. Athletic trainers value the service of being able to minimize their patient’s pain and improve overall physical comfort. Participants within viewpoint 1 placed high importance on the fact that “athletic trainers have the skills and knowledge to provide quality healthcare to varied patient populations” (st. 33, factor ranking = 4, Z score = 1.51) and “athletic trainers address pain management” (st. 8, factor ranking = 4, Z score = 1.49). The statement “athletic trainers work as a team in care delivery to patients” (st. 15, factor ranking = 3, Z score = 1.19) was also ranked high in viewpoint 1 compared to the other group of healthcare professionals, accompanied by open-ended statements explaining the benefits of interprofessional healthcare teams. Athletic trainers highly valued the statement of “open and effective communication between patients and athletic trainers occurs” (st. 32, factor ranking = 3, Z score = 1.15) and even further exhibited value in interpersonal tendencies by also ranking the statement “athletic trainers involve patients in decisions about their care” (st. 5, factor ranking = 3, Z score = 1.14) within the top six statements seen on the composite Q-sort. Participants within this viewpoint ranked the statement “athletic trainers integrate the ICF model as a framework for delivery of patient care” (st. 4, factor ranking = -5, Z score = -2.13) as the one statement they disagreed with the most, simply due to lack of knowledge about the ICF model. Table 1 includes open-ended responses from participants respective to the top six highest ranked statements.

**Table 1:** Viewpoint 1 open-ended statements.

Viewpoint 1: The Interpersonal Connection
Statement 1: *“Every athlete that comes into our clinic for help deserves respect*, *period*. *We are all human beings*. *If we treat every person with dignity and respect then they are more likely to be honest about their injury*, *trusting us and returning for other injuries*.*”* (38-year-old female in the secondary school setting with 13 years of experience)
Statement 33: *“I personally have seen a variety of patients and of all walks of life and I have learned to adapt my approach and increase my knowledge to accommodate a wide variety of patient populations*. *I feel often too that in many degrees this is under looked and under appreciated by our health care system*.*”* (35-year-old male in the independent contractor setting with 5 years of experience)
Statement 8: *“Pain is the biggest reason for the patients to start looking for a healthcare provider*.*”* (35-year-old male in the professional sports setting with 6 years of experience)
Statement 15: *“I have felt that the medical professionals I have worked with have done an excellent job of consulting one another and providing care as a team*. *Everyone has more experience in one field or area of the body than another and we have been effective at working as a team to provide the best care possible*.*”* (25-year-old male in the professional sports setting with 1 year of experience)
Statement 32: *“Regardless of what setting or clinical affiliation I have had in the past*, *and with my personal career*, *I am HUGE on communication*. *I have yet to see an athletic trainer not have open communication with their patients regarding all matters of their care*. *The beauty of this profession we truly get to meet our "patients" as people before an injury when we need to call them patients*.*”* (26-year-old female in the occupational health setting with 2 years of experience)
Statement 5: *“We definitely do our best to make sure the patient’s voice is heard and incorporate their preferences as much as we are able*.*”* (28-year-old female in the independent contractor setting with 6 years of experience)

### Viewpoint 2: The holistic gatekeeper

Health care providers grouped into viewpoint 2 also aligned with the core principles of patient preferences, and information and education but demonstrated those viewpoints with a few different ranked statements. The highest ranked statement for this viewpoint was statement 2: “athletic training is focused on improving patients’ quality of life” (st. 2, factor ranking = 5, Z score = 2.13). Athletic trainers within this viewpoint found value in looking out for the patient’s health in the long term, as well as deemed it crucial for patients to gain their independence and autonomy back in sports specific and daily living needs before their care was considered complete. Open-ended responses from participants within this viewpoint highlighted the value ATs have for PCC and how it tailors their decisions as a clinician regarding patient goals. Similar to viewpoint 1’s composite Q-sort, healthcare providers grouped in viewpoint 2 found high value in the statements of “athletic trainers have the skills and knowledge to provide quality healthcare to varied patient populations” (st. 33, factor ranking = 4, Z score = 1.86) and “athletic trainers treat patients with dignity and respect” (st. 1, factor ranking = 4, Z score = 1.56). The statements “athletic trainers consider patients daily living needs” (st. 9, factor ranking = 3, Z score = 1.15) and “athletic trainers support and educate patients on autonomy and self-care” (st. 31, factor ranking = 3, Z score = 1.14) were ranked very close on the composite Q-sort and both promote the overall patient independence and quality of life that healthcare providers in this viewpoint strive for. Participants within this viewpoint ranked the statement “appointment scheduling is easy” (st. 21, factor ranking = -5, Z score = -1.67) as the one they disagreed with the most. Table 2 includes open-ended responses from participants respective to the top six highest ranked statements.

**Table 2:** Viewpoint 2 open-ended statements.

Viewpoint 2: The Holistic Gatekeeper
Statement 2: *“As healthcare professionals*, *the goal is to view the patient as a person and not just their injury to be able to provide the best care possible to improve the patients’ quality of life in all aspects*, *if possible*, *from work life*, *home life and all other qualities that are important/fulfilling to the patient*. *This approach assists in helping the patient recover as a whole and not just recover from their injury/condition to allow the patient to improve their quality of life in which athletic trainers can assist the patient with through various physical/holistic rehabilitation approaches*.*”* (26-year-old female in the occupational health setting with 4 years of experience)
Statement 33: *“I believe that the skillset and preparation of Athletic Trainers is incredibly valuable and allows for practitioners to make a significant difference in the lives of a wide variety of patients in a broad array of healthcare concerns*. *Standardization of the certification process*, *credentialing*, *and continuing education requirements set a baseline expectation for quality of the profession*.*”* (34-year-old male in the military/law enforcement/government setting with 12 years of experience)
Statement 1: *“Patient focused*, *quality healthcare is one of the primary values of my organization and the athletic training profession as a whole*. *Our patients’ well-being and quality of life should always be our top priority*.*”* (28-year-old male in the secondary school setting with 6 years of experience)
Statement 9: “*The patient’s daily activities are important moving to a healthy life*, *on and off the sport pitch*.*”* (43-year-old male in the amateur/recreational/youth sports setting with 14 years of experience)
Statement 31: *“I truly believe patient needs to be independent upon discharge*.*”* (36-year-old male in the hospital setting with 6 years of experience)
Statement 5: *“I think the best practice is when patients have autonomy in their care*. *If the treatment is patient-centered*, *it will be tailored specifically to their issues whether it be physical and/or mental/emotional and they will have greater buy-in to the plan*.*”* (26-year-old female in the professional sports setting with 4 years of experience)

## Discussion

Healthcare provider perspective regarding PCC is important to collect and compare across numerous professions to analyze similarities, differences, and overall dimensions that need improvement. The purpose of this research study was to determine how ATs place importance on the principles of PCC. To our knowledge, there are no studies published in athletic training scholarly outlets using Q methodology. Two distinguishing viewpoints were found through factor analysis of the Q-sorts: 1) the interpersonal connection that valued teamwork, open communication, and respectful care with varied populations; 2) the holistic gatekeeper that valued personal promotion for activities of daily living, self-care, and quality of life. Participants were divided into these two groups so that athletic trainers placed in viewpoint 1 could not also be placed into viewpoint 2. A common theme found across both viewpoints was that ATs placed high values on patient’s preferences, as well as the core principle of information and education shared between patient and healthcare provider. Dignity and respect were a top priority for most ATs across their Q-sorts, while knowledge of the ICF model as a framework for their athletic training practice was seen as a low priority simply because most participants do not know what it was.

### Patient-centered care in healthcare

Patient-centered care is not a new focal point in healthcare, and various principles have served as driving forces in how healthcare professionals interact with their patients for years [10]. Just as medicine has evolved, the Golden Rule of “do unto others as you would have them do unto you” has slowly been phased out and been replaced by the newer “Platinum Rule,” which states “treat people the way they want to be treated, rather than how you would want to be treated” [24]. As a much needed upgrade of an outdated cultural principle, the Platinum Rule fits the definition of PCC seamlessly and directly incorporates the dimension of patient preferences into patient-provider interactions. The findings from our study and recent literature on the Platinum Rule suggest a positive alternative in PCC that needs further exploration in different healthcare settings [25, 26].

Previous research looking at a range of hospital staff and their viewpoints and values of PCC found that both job setting and overall patient population had a large impact on how participants ranked and valued their PCC statements [7]. Subgroup analysis of two different hospital departments found that participants who work in the surgical intensive care unit valued continuity of care and the ability to have the appropriate records transferred along with the patient to their next unit, while those that worked with a more geriatric population in another department valued the ability to have a primary contact for the patient who knows everything about their medical history [7]. Our study discovered that Ats across various job settings and patient populations had differing viewpoints of PCC and valued some dimensions more than others. Both viewpoints valued the core principle of patient preferences the strongest, seen through the selection of their highest-ranking statements. The viewpoints demonstrated differing preferences for the principles of information and education, coordination of care, and physical comfort across their respective composite Q-sort. Based on these results, Ats across the board believe they hold their patients’ preferences as a top priority in overall clinical decision-making and care. However, unless the AT treating the patient know how they want to be treated, they will not be implementing PCC to the new “Platinum Rule” standards.

### Dignity & respect

Previous research exploring PCC principles from healthcare providers in geriatric and surgical intensive care hospital units expressed a common viewpoint of “treating patients with dignity and respect” [7]. The findings in the prior study considered this statement a foundational building block to every other aspect of healthcare and also placed high importance on involving patients in decisions regarding their plan of treatment and care [7]. Similarly within our study, “athletic trainers treat patients with dignity and respect” (st. 1) was a statement that ranked highest in distinguishing viewpoint 1 as the most valued spot on the Q-sort (factor ranking = 5) and tied for second highest in distinguishing viewpoint 2 (factor ranking = 4), indicating that a majority of the participants value these two traits in their daily clinical practice when interacting with patients. Dignity and respect within the statements fall under the core principle of patient preferences. Although confidence in one’s ability to implement PCC is important, it is different from successfully performing PCC in a real clinical setting, which has been identified in athletic training as a weakness in emergency care skills and healthcare administration. Athletic training literature has recently started to address the need to measure providers’ application of PCC throughout their care by asking the very patients Ats interact with daily [27]. When surveyed on how well Ats embody PCC, student-athletes demonstrated strong agreement with the statement that Ats at their college/university delivered care that was respectful of their preferences [27]. These patient findings are significant and match the viewpoints that Ats in our study had for themselves regarding PCC, but more research needs to be done comparing perceptions across all athletic training clinical settings and patient populations.

### Provider engagement with patients

While ranked statement similarities were shared between the two viewpoints, the main difference between the Ats is what constituted an ideal patient-provider relationship in relation to PCC concepts. The top statement for viewpoint 1 was centered around dignity and respect for the patient, as well as followed by other statements focused on what the provider can do for the patient and how they can best serve them. The Ats within this viewpoint are focused on a relationship that allows the provider to complete services for the patient. Viewpoint 2 had a vastly different top ranked statement; “athletic training is focused on improving patients’ quality of life” (st. 2) and incorporated with the 5 other statements identified that Ats within this viewpoint were focused on preparing the patients to be autonomous in their daily lives. The Ats within viewpoint 2 believed in patient engagement, yet highlighted that PCC was rooted in personal promotion and self-care. The Ats that expressed a strong connection to viewpoint 2 seemed to be focused on social and mood support which aligns with previous literature on the role of Ats following injuries [28]. As a core principle of PCC, actively involving patients in decisions about their own medical care, treatment, and overall goals is crucial to a healthy patient-provider bond and successful patient compliance [2]. Future research should consider exploring how the two distinct viewpoints, providing the best possible care and promoting patient autonomy, influence patient satisfaction and outcomes within athletic training job settings.

### ICF model

Athletic trainers across our study did not rank the ICF model as a guiding principle for PCC and ranked it low on their Q-sorts. Some participants did not know what the ICF model is and because of this lack of knowledge do not incorporate it into their daily clinical practice. Multiple open-ended responses from participants ranked “athletic trainers integrate the ICF model as a framework for delivery of patient care” (st. 7) as their least agreed with statement (factor ranking = -5) because they had never heard of it before. This is concerning as the ICF framework has been officially adopted as a healthcare delivery framework by the NATA Board of Directors since 2015; however, it has only recently been adopted into the curriculum standards for professional-level athletic training education creating a potential knowledge-to-practice gap [29]. The ICF model is used to identify and address patient barriers to access healthcare and treatment as well as to allow healthcare providers an opportunity to successfully tailor care to the patient’s needs [30]. If ATs do not know what the ICF framework is in the first place, they cannot be expected to utilize it as a method to determine clinical practice decisions and promote evidence-based medicine. In order to reach those in the profession with a lack of knowledge for the ICF framework, continuing education on PCC may need to be considered for ATs to have a chance to fully understand and incorporate those tactics into their clinical practice. Athletic trainers across job settings had differing viewpoints of PCC. The distinguishing PCC viewpoints were established around patient preferences, and information and education. Moreover, ATs value dignity and respect of their patient; yet, the ICF Framework had a universal lack of importance amongst the participants relative to PCC. Now that the framework for determining if and how ATs value PCC and its eight principles in their clinical practice has been determined, more research should be completed exploring how ATs actively incorporate the PCC principles when interacting with their patients and if the patients believe their healthcare providers are successfully implementing it.

### Limitations & future research

The study integrated a list of PCC statements from other healthcare providers used in previous research. One concern with Q methodology is that post-hoc validation and reliability assessments are not practical nor possible due to the qualitative nature of the research. We suggest future researchers explore validating a comprehensive list of tasks, skills, and objectives for PCC that can be used comprehensively throughout medicine and healthcare. A limitation found by this study was the novelty of Q methodology parameters for the participants of the study, as many were unfamiliar with the process of ranking statements across a Q-sort. Many participants believed that they were not able to rank their statements in correct locations on the board based on the ratio of agree, disagree, and neutral statements. Instructions were provided at the beginning of the study, as well as during the Q-sort process to support participants with any issues they might have had while navigating through the survey. Q methodology, while a novel and unique research technique, provides researchers with the opportunity to gain qualitative and quantitative data on participants’ values and opinions [19].

A limitation could have also stemmed from the sample size of our study, which may have affected the study’s findings. With a total of 166 respondents from undetermined educational experiences, whether that be an accredited entry-level or post-professional program or continuing education requirements, the current findings may not be generalizable to all athletic trainers. In addition, the authors feel it is imperative to note that the study was executed from April-May 2020 during the height of the COVID-19 pandemic. Therefore, the timing of data collection may have resulted in fewer responses or decision fatigue relative to the concept. Future research should also address aspects of PCC that athletic training is lacking in, such as access to care and the process of involving patients in care decision making.

## Conclusion

Athletic trainers’ perceptions and viewpoints of PCC were quantitatively and qualitatively collected to establish the healthcare providers’ values and preferences towards the 8 principles of PCC. Two distinguishing PCC viewpoints surfaced from the pool of ATs after factor analysis and providers within our study exhibited that they value patients’ preferences, information and education. The findings are critical to the future exploration on patient satisfaction and the delivery of PCC in clinical settings. In addition, the ICF model, which is an adopted framework by the NATA, was identified to have a universal lack of importance amongst ATs in our study. This information helps to inform our profession on the perceptions held by ATs relative to what they believe they are doing is PCC. Finally, the data reveals a contrast between the viewpoints held by ATs and the definition in the literature.

## Supporting information

S1 Table Patient-centered care 36-item statement list. (DOCX)

S2 Table Additional open-ended responses. (DOCX)

S1 File (CSV)

S2 File (CSV)

S3 File (XLSX)

S4 File (CSV)

## References

[1] Berwick DM, Nolan TW, Whittington J. The triple aim: Care, health, and cost. Health Aff. 2008;27(3):759–69. 10.1377/hlthaff.27.3.759 18474969

[2] Mead N, Bower P. Patient-centredness: A conceptual framework and review of the empirical literature. Social science & medicine (1982). 2000;51(7):1087–110. doi: 10.1016/s0277-9536(00)00098-8 11005395

[3] Kitson A, Marshall A, Bassett K, Zeitz K. What are the core elements of patient-centred care? A narrative review and synthesis of the literature from health policy, medicine and nursing. Journal of advanced nursing. 2013;69(1):4–15. doi: 10.1111/j.1365-2648.2012.06064.x 22709336

[4] Mohammed K, Nolan MB, Rajjo T, Shah ND, Prokop LJ, Varkey P, et al. Creating a patient-centered health care delivery system: A systematic review of health care quality from the patient perspective. American journal of medical quality: the official journal of the American College of Medical Quality. 2016;31(1):12–21. doi: 10.1177/1062860614545124 25082873

[5] Brummel-Smith K, Butler D, Frieder M, Gibbs N, Henry M, Koons E, et al. Person-centered care: A definition and essential elements. Journal of the American Geriatrics Society. 2016;64(1):15–8. doi: 10.1111/jgs.13866 26626262

[6] Corazzini KN, Lekan-Rutledge D, Utley-Smith Q, Piven ML, Colon-Emeric CS, Bailey D, et al. "The Golden Rule": Only a starting point for quality care. Director (Cincinnati, Ohio). 2005;14(1):255–93. PMC1636677 17334452

[7] Berghout M, van Exel J, Leensvaart L, Cramm JM. Healthcare professionals’ views on patient-centered care in hospitals. BMC health services research. 2015;15:385. doi: 10.1186/s12913-015-1049-z 26373841 PMC4572638

[8] Institute of Medicine (US) Committee on Quality of Health Care in America. Crossing the quality chasm: A new health system for the 21st Century. Washington (DC): National Academies Press (US). 2001. 25057539

[9] Davis K, Schoenbaum SC, Audet AM. A 2020 vision of patient-centered primary care. Journal of general internal medicine. 2005;20(10):953–7. doi: 10.1111/j.1525-1497.2005.0178.x 16191145 PMC1490238

[10] Rathert C, Wyrwich MD, Boren SA. Patient-centered care and outcomes: A systematic review of the literature. Medical care research and review: MCRR. 2013;70(4):351–79. doi: 10.1177/1077558712465774 23169897

[11] Epstein RM, Franks P, Fiscella K, Shields CG, Meldrum SC, Kravitz RL, et al. Measuring patient-centered communication in patient-physician consultations: theoretical and practical issues. Social science & medicine (1982). 2005;61(7):1516–28. doi: 10.1016/j.socscimed.2005.02.001 16005784

[12] Bensing J. Bridging the gap: The separate worlds of evidence-based medicine and patient-centered medicine. Patient education and counseling. 2000;39(1):17–25. 11013544 10.1016/s0738-3991(99)00087-7

[13] Institute for Patient- and Family-Centered Medicine. Advancing the Principles of Patient-Centered Care 2013. https://www.ipfcc.org/resources/picker-institute.html.

[14] Paessler HH. Input of second opinion in orthopedic sports medicine. Springer Berlin Heidelberg. 2014. 3171–80 p.

[15] Baugh CM, Kroshus E, Lanser BL, Lindley TR, Meehan WP JJoat. Sports medicine staffing across National Collegiate Athletic Association Division I, II, and III schools: evidence for the medical model. 2020;55(6):573–9. 10.4085/1062-6050-0463-19 PMC7319741 32364760

[16] Redinger AS, Winkelmann ZK, Eberman LE. Collegiate student-athletes’ perceptions of patient-centered care delivered by athletic trainers. J Athl Train. 2021;56(5):499–507. doi: 10.4085/130-20 33150412 PMC8130768

[17] Epstein RM, Fiscella K, Lesser CS, Stange KC. Why the nation needs a policy push on patient-centered health care. Health Aff. 2010;29(8):1489–95. 10.1377/hlthaff.2009.0888 20679652

[18] Kelly SE, Moher D, Clifford TJ. Expediting evidence synthesis for healthcare decision-making: exploring attitudes and perceptions towards rapid reviews using Q methodology. Peer J. 2016;4:e2522. doi: 10.7717/peerj.2522 27761324 PMC5068451

[19] Valenta AL, Wigger U. Q-methodology: Definition and application in health care informatics. Journal of the American Medical Informatics Association: JAMIA. 1997;4(6):501–10. doi: 10.1136/jamia.1997.0040501 9391937 PMC61268

[20] Ho GW. Examining perceptions and attitudes: A review of Likert-type scales versus Q-metholody. West J Nurs Res. 2017;39(5):674–89. 27456460 10.1177/0193945916661302

[21] Watts S, Stenner P. Doing Q Methodological Research: Theory, Method and Interpretation. London: SAGE Publications Ltd; 2012 2021/03/29.

[22] Brown SR. A primer on Q methodology. Operant Subjectivity. 1993;16.

[23] Lutfallah S, Buchanan L. Quantifying subjective data using online Q-methodology software. The Mental Lexicon. 2019;14(3):415–23.

[24] Christensen L, Medina A. Informing the bent golden rule or reforming it–the platinum rule. Social Studies Research and Practice. 2016;11:92–2016.

[25] Chochinov HM. The Platinum Rule: A New Standard for Person-Centered Care. Journal of Palliative Medicine. 2022. doi: 10.1089/jpm.2022.0075 35230173 PMC9145569

[26] Chochinov HM, McClement S, Hack T, Thompson G, Dufault B, Harlos M. Eliciting personhood within clinical practice: effects on patients, families, and health care providers. Journal of pain symptom management. 2015;49(6):974–80. e2. doi: 10.1016/j.jpainsymman.2014.11.291 25527441

[27] Redinger AS, Winkelmann ZK, Eberman LE. Collegiate student-athletes’ perceptions of patient-centered care delivered by athletic trainers. Journal of athletic training. 2020;Online Early. 10.4085/130-20 PMC8130768 33150412

[28] Yang J, Schaefer JT, Zhang N, Covassin T, Ding K, Heiden E. Social support from the athletic trainer and symptoms of depression and anxiety at return to play. J Athl Train. 2014;49(6):773–9. doi: 10.4085/1062-6050-49.3.65 25329346 PMC4264649

[29] Comission on Accreditation of Athletic Training Education. 2020 Standards for Accreditation of Professional Athletic Training Programs. Master’s Degree Programs. Austin, TX: CAATE; 2018. p. 22.

[30] Nottingham S, Meyer C, Blackstone B. ICF Model: A framework for athletic training practice: National Athletic Trainers’ Association; 2016 https://www.nata.org/blog/beth-sitzler/icf-model-framework-athletic-training-practice.

