# Supplementary material for: Athletic trainers’ viewpoints of patient-centered care: Preliminary findings
Source: PLoS One. 2022 Sep 14;17(9):e0274577. doi: 10.1371/journal.pone.0274577 (PMC9473394; doi:10.1371/journal.pone.0274577)
Supplement: S1 Table — (DOCX) [file pone.0274577.s001.docx]

**S1 Table. Patient-Centered Care 36-Item Statement List.**

| **Patient-Centered Care Principle** | **Patient-Centered Care Objective** | **Statement** |
| --- | --- | --- |
| **Patients’ preferences** | Providing care in a respectful atmosphere with dignity and respect | 1. Athletic trainers treat patients with dignity and respect. |
|  | Focus on quality-of-life issues / whole-person care | 1. Athletic training is focused on improving patients’ quality of life. |
|  |  | 1. Athletic trainers take patients’ preferences into account. |
|  |  | 1. Athletic trainers integrate the International Classification of Functioning, Disability, and Health (ICF) model as a framework for delivery of patient care. |
|  | Informed and shared decision making | 1. Athletic trainers involve patients in decisions about their care. |
|  | Personal goals and outcomes | 1. Patients are supported in setting and achieving their own treatment goals. |
|  |  | 1. Athletic trainers consider the social determinants of health when planning care. |
| **Physical comfort** | Pain management | 1. Athletic trainers address pain management. |
|  | Assistance with daily living needs | 1. Athletic trainers consider patients’ daily living needs. |
|  | Surroundings and environment | 1. Patient areas in the healthcare facility are clean and comfortable. |
|  |  | 1. Athletic trainers ensure privacy in the healthcare facility. |
| **Coordination of care** | Coordination and integration of care | 1. Athletic trainers are well informed; patients need to tell their story only once. |
|  |  | 1. Patient care is well coordinated among the healthcare professionals. |
|  | Spokesperson for navigation through the system | 1. Patients have a primary contact who knows everything about their condition and treatment. |
|  | Teamwork | 1. Athletic trainers work as a team in care delivery to patients. |
| **Emotional support** | Anxiety about consequences of the changed situation | 1. Athletic trainers recognize a patients’ anxiety about their situations. |
|  | Creating support systems | 1. Athletic trainers involve the patient’s social support system to assist their emotional response. |
|  | Anxiety about the impact of one’s illness on one’s family and loved ones | 1. Athletic trainers pay attention to the patients’ anxiety about the impact of their injury/illness on their social support system. |
| **Access to care** | Access to location/specialist | 1. The healthcare facility is accessible for all patients. |
|  | Availability of transportation | 1. Clear directions are provided to and inside the healthcare facility. |
|  | Clear instructions provided on how and when to get referral |  |
|  | Ease of scheduling appointments | 1. Appointment scheduling is easy. |
|  | Waiting time | 1. Waiting times for appointments are acceptable. |
|  | Language barrier | 1. Language is not a barrier to access to care. |
|  | Cultural differences | 1. Athletics trainers provide education and materials respective to one’s health literacy level. |
| **Continuity and transition** | Understandable, detailed information regarding all aspects of care | 1. When a patient is transferred to another healthcare facility or professional, relevant patient information is also transferred. |
|  | Coordination and planning of ongoing treatment | 1. Patients who are transferred are well informed about where they are going, what care they will receive, and who their contact person will be. |
|  | Provide information regarding access to support after hospital discharge | 1. Patients receive skilled advice before discharge. |
|  |  | 1. Athletic trainers collect information through patient-reported outcome measures to inform their care decisions. |
| **Information and education** | Information on all aspects of care (e.g., clinical status, progress, prognosis, care processes) | 1. Patients are well informed about all aspects of their condition and care (e.g., clinical status, progress, and prognosis). |
|  | Information on processes of care | 1. Patients can access their medical records. |
|  | Information and education to facilitate autonomy and self-care | 1. Athletic trainers support and educate patients on autonomy and self-care. |
|  | Open communication between patient and caregiver | 1. Open and effective communication between patients and athletic trainers occurs. |
|  | Skills and knowledge of caregiver | 1. Athletic trainers have the skills and knowledge to provide quality healthcare to varied patient populations. |
| **Family and friends** | Accommodations | 1. Athletic trainers acknowledge the role of a social support system in the care of the patient. |
|  | Support for family as caregivers | 1. Athletic trainers connect the social support system that the patient relies upon with necessary resources in or near the healthcare facility. |
|  | Respect for role in decision making | 1. Athletic trainers involve the patient’s social support system in decisions about the patient’s care. |
