# Supplementary material for: Athletic trainers’ viewpoints of patient-centered care: Preliminary findings
Source: PLoS One. 2022 Sep 14;17(9):e0274577. doi: 10.1371/journal.pone.0274577 (PMC9473394; doi:10.1371/journal.pone.0274577)
Supplement: S2 Table — (DOCX) [file pone.0274577.s002.docx]

**S2 Table. Additional Open-Ended Responses**.

| PCC Statement | Lowest Statement Reasoning | Highest Statement Reasoning |
| --- | --- | --- |
| 1. Athletic trainers treat patients with dignity and respect. | 0, 0% | “Improving quality of life and maintaining it for the long term is what an athletic trainer is essentially in place for no matter the setting.” (43-year-old male in the hospital setting with 20 years of experience)  “I believe that what sets athletic trainers apart from most healthcare professionals is the relationships that grow between clinician and patient and that helps us treat each patient with dignity and respect and that helps further grow the patient's trust in us.” (25-year-old female in the military/law enforcement/government setting with 4 years of experience) |
| 1. Athletic training is focused on improving patients’ quality of life. | “Athletic training consistently prioritizes return to play rather than an overall return to health and function.” (27-year-old female in the professional sports setting with 2 years of experience) | “Quality is an important concept to what we do. Population health will be at the forefront of healthcare, and we are population health experts. Quality of life is an important component of population health, and we strive to improve quality of life with every patient we see.” (35-year-old male working in the occupational health setting with 11 years of experience)  “Quality of life is an all-encompassing statement and uniquely individual to each patient; when I'm working one-on-one with a patient, I'm not addressing a hamstring strain, I'm addressing a police officer or a recruit with posterior thigh pain who is unable to complete a foot pursuit, so he loses his part time shifts and is also a new dad who's unable to bend down and pick up his daughter. I consider all of these when I am working with a patient and trying to improve their overall quality of life through a variety of physical & psychosocial interventions.” (26-year-old female in the military/law enforcement/government setting with 5 years of experience). |
| 1. Athletic trainers take patients’ preferences into account. | 0, 0% | “In my experiences, to maximize the patient’s outcome, the patient’s preferences on treatments, exercises, or goal setting allow for better patent compliance, participation, and ultimately a successful return to play.” (46-year-old male in the professional sports setting with 24 years of experience) |
| 1. Athletic trainers integrate the International Classification of Functioning, Disability, and Health (ICF) model as a framework for delivery of patient care. | “I do not know what this is, and therefore it doesn't play a role in my practice.” (25-year-old male in the professional sports setting with 4 years of experience)  “I don't think that most athletic trainers know what the ICF is.” (43-year-old male in the hospital setting with 20 years of experience)  “I am unaware of the ICF process.” (25-year-old female in the other setting with 3 years of experience)  “I have never used [the ICF Model] as a clinician, in fact I've never heard of it in any continuing education I have done.” (29-year-old female in the college/university setting with 7 years of experience) | 0, 0% |
| 1. Athletic trainers involve patients in decisions about their care. | 0, 0% | “Athletic trainers try to work with all sorts of demographics and try to communicate and work with them to facilitate the best care that works for them. Often being more informative than the doctors, and hospitals.” (55-year-old female in the occupational health setting with 31 years of experience) |
| 1. Patients are supported in setting and achieving their own treatment goals. | “Goals are set for them based on assessed needs and MD orders.” (32-year-old male in the clinic setting with 30 years of experience) | 0, 0% |
| 1. Athletic trainers consider the social determinants of health when planning care. | “When we are treating an athlete for an injury, could be life threatening, we need to get them the care they need. If it is an emergency, they need the kind of care to save their life; social determinants are not what are going through my mind. I want the body part treated and worry about the other factors later.” (38-year-old female in the secondary school setting with 13 years of experience) | “You have to determine all aspects of the athlete’s life when you are taking care of their health and well-being.” (27-year-old female in the secondary school setting with 5 years of experience) |
| 1. Athletic trainers address pain management. | 0, 0% | “Because that is what we do... we address pain management and build our rehabilitation programs.” (37-year-old female in the college/university setting with 16 years of experience) |
| 1. Athletic trainers consider patients’ daily living needs. | 0, 0% | “Athletic trainers always look at daily needs of patients as first priority.” (35-year-old female in the occupational health setting with 13 years of experience) |
| 1. Patient areas in the healthcare facility are clean and comfortable. | “Yes.” (24-year-old female in the college/university setting with 1 year of experience) | “It has been a very rare circumstance that I've seen healthcare facilities without appropriate accommodating and cleanly offices.” (29-year-old female in the clinic setting with 6 years of experience) |
| 1. Athletic trainers ensure privacy in the healthcare facility. | “I believe the space and daily workings of athletic training facilities often do not allow for appropriate privacy. Delivering healthcare or injury news often happens when other patients are on adjacent tables, for example.” (37-year-old female in the college/university setting with 15 years of experience) | “Ensuring athlete/patient privacy in any setting should be the first thing in an athletic trainer's mind.” (24-year-old male in the professional sports setting with 3 years of experience) |
| 1. Athletic trainers are well informed; patients need to tell their story only once. | “I believe there is no limit to as many times as a patient should tell "their story". Each time the story is told new details may surface, whether it be an emotional piece or another physical detail. Patients will often say, "I forgot about that". I think repeating the story will eventually get all the pieces of the puzzle that is their issue.” (26-year-old female in the professional setting with 4 years of experience) | 0, 0% |
| 1. Patient care is well coordinated among the healthcare professionals. | “Patient Care is well coordination among healthcare professionals.” (51-year-old male in the amateur/recreational/youth sports with 28 years of experience) | “Patient care is paramount.” (32-year-old male in the occupational health setting with 10 years of experience) |
| 1. Patients have a primary contact who knows everything about their condition and treatment. | “Not every patient wants to involve others in their personal care. If they invite others in such as parents or a close friend, I am happy to bring them up to speed. However, the patient is my focus unless they are unable to comprehend important information and decisions.” (55-year-old male in the hospital setting with 30 years of experience)  “Patients who are seeing multiple doctors do not always know their diagnosis same with their immediate support system. Often patients believe that their full health history is non-essential when treating certain injuries and diagnosis and leave this out when providing information at the beginning of treatment.” (26-year-old female in the other setting with 2 years of experience) | “I believe one role athletic trainers fill that other healthcare professionals may not is the availability of daily contact with patients or student-athletes. This allows for a free flow of information and history understanding. Often times, an athletic trainer that has worked with someone for 4 years straight is able to rattle off their health history without chart review.” (37-year-old female in the college/university setting with 15 years of experience) |
| 1. Athletic trainers work as a team in care delivery to patients. | “In my experience Athletic Trainers do not always reach-out for help and assistance. They have been taught that we are supposed to work a lot of hours and its part of the job, so they work really hard, and rarely ask for assistance in things. We have so many skills, and knowledge, and yes, we CAN do therapy and return athletes back to participation safely, but do we actually have the time, and can we dedicate the time to each person, so they get the "quality" of care they deserve?” (37-year-old female in the secondary setting with 19 years of experience) | “Athletic trainers are very much part of a team that provides care to athletes and athletic populations. As an athletic trainer, we work under a supervising physician. In my setting, I work closely with a full orthopedic clinic, surgical hospital, dentist, physical therapists, chiropractors, as well as a strength and conditioning coach. We are all part of the larger team and organization.” (29-year-old male in the professional sports setting with 6 years of experience) |
| 1. Athletic trainers recognize a patients’ anxiety about their situations. | 0, 0% | “In my opinion the mental health of athletes/patients is often an overlooked component of any injury/illness. As patients start to think about their condition and the impact of it, they can often send themselves into a downward spiral. As the front line of healthcare, it is an important role of the athletic trainer to manage not only the musculoskeletal side of the patient, but also their mental wellbeing. Too much communication between patient and clinician is not a thing. A lot of uneasiness in patients comes from not being well informed enough about pathology, treatment, and prognosis. One of my main goals during any eval., treatment session, or anything of the like is to walk the patient through it and explain everything that I am doing with them. Open communication leads to better outcomes in my experience. Hopefully, all healthcare employees will keep this in mind when working with their respective populations.” (22-year-old male in the secondary school setting with 1 year of experience) |
| 1. Athletic trainers involve the patient’s social support system to assist their emotional response. | “I don't see the importance of involving social life into patient outcomes.” (32-year-old male in the occupational health setting with 10 years of experience) | “Yes.” (24-year-old female in the college/university setting with 1 year of experience) |
| 1. Athletic trainers pay attention to the patients’ anxiety about the impact of their injury/illness on their social support system. | 0, 0% | 0, 0% |
| 1. The healthcare facility is accessible for all patients. | “The healthcare system is not available to all of my patients because some of my patients do not have access to health insurance for various reasons; and, therefore, these patients are unable to afford the healthcare recommended for return to sport after various injuries/illnesses/conditions.” (28-year-old male in the secondary school setting with 6 years of experience) | “In my experience, all buildings that I have been working in have been to federal/state code. Now, are they up to healthcare standards, probably not.” (48-year-old male in the military/law enforcement/government setting with 21 years of experience)  “It is my belief, in some professional settings, the TR is not all ways available to younger (rookie) athletes or preventative type treatments. Our facility is available to all.” (40-year-old male in the professional setting with 19 years of experience) |
| 1. Clear directions are provided to and inside the healthcare facility. | “Many Athletic Trainers do not have contact with patients prior to initial contact or often do not know who is coming in and when. Unless someone reaches out for specific directions, usually no communication is given for finding the clinic. Signage can be variable.” (34-year-old male in the military/law enforcement/government setting with 12 years of experience) | 0, 0% |
| 1. Appointment scheduling is easy. | “Depending on the patient's insurance, I think that getting an appointment with the appropriate physician/practitioner can be too difficult and the process complicated.” (28-year-old female in the independent contractor setting with 3 years of experience)  “In my high school setting, I don't have scheduled appointments, athletes walk in when they need to or are told to by their coaches.” (27-year-old female in the secondary school setting with 5 years of experience) | 0, 0% |
| 1. Waiting times for appointments are acceptable. | “Long wait times are not acceptable. Emergencies are different. But just to see someone and to sit and wait is no way to treat a customer. Know how you work and schedule accordingly.” (52-year-old male in the secondary school setting with 28 years of experience)  “When seeing patients in the office it is normal to run behind especially when they need to see orthopedic physicians.” (23-year-old female in the amateur/recreational/youth sports setting with 1 year of experience) | “My patients have almost no wait times.” (26-year-old female in the occupational health setting with 3 years of experience) |
| 1. Language is not a barrier to access to care. | “Language barriers are absolutely a large barrier in the athletic training environment. Whether English is a second language, or for English speakers, if we are not putting terms into plain English and not speaking in "medical jargon" it can be difficult for our patients to understand us.” (31-year-old female in the military/law enforcement/government setting with 10 years of experience)  “I am Japanese and know a lot of athletes whose first language is not English. For those it can be a huge barrier to communicate especially if it comes into medical terminology.” (35-year-old male in the professional sports setting with 6 years of experience) | “We have bilingual staff members, so language is not an issue.” (40-year-old female in the professional sports setting with 18 years of experience) |
| 1. Athletics trainers provide education and materials respective to one’s health literacy level. | “Literacy level not an issue with population I work with.” (47-year-old male in the military/law enforcement/government setting with 25 years of experience) | “I think education of athletes is important. Giving patients the information, they need and can do in order to stay healthy and do things on their own is very important. Especially when we are stretched so thin with the number of athletes we care for daily.” (37-year-old female in the secondary school setting with 19 years of experience) |
| 1. When a patient is transferred to another healthcare facility or professional, relevant patient information is also transferred. | “If the situation is not related to a work issue, then no information is transferred unless the patient communicates them self.” (37-year-old female in the occupational health setting with 15 years of experience) | “Communication is key among health care professionals and making sure the athletes know what is going on with their treatment and care is paramount.” (35-year-old male in the college/university setting with 12 years of experience) |
| 1. Patients who are transferred are well informed about where they are going, what care they will receive, and who their contact person will be. | “Most other health care systems do not understand who or what an athletic trainer is so connecting with them is almost impossible in the industrial setting and panel physicians and organizations changing all the time. There is no consistency to build a relationship. They do not want to.” (55-year-old female in the occupational health setting with 31 years of experience)  “Due to the rapidly changing medical staff in the region as well as the multiple facilities patients are transferred to, consistency is sometimes a problem.” (28-year-old female in the independent contractor setting with 6 years of experience) | 0, 0% |
| 1. Patients receive skilled advice before discharge. | 0, 0% | “I believe I speak to all athletes before we let them start to practice or are released back to an active life.” (44-year-old male in the secondary school setting with 18 years of experience) |
| 1. Athletic trainers collect information through patient-reported outcome measures to inform their care decisions. | “In my setting, it is important to get the athlete back to playing condition and the athletes are happier and report good outcomes if they get back to playing. Therefore, I do not see the value of patient reported outcomes in my setting.” (24-year-old male in the professional sports setting with 3 years of experience)  “Patient does not always be truthful about their outcomes.” (67-year-old male in the independent contractor setting with 40 years of experience) | “I think athletic trainers do a great job of collecting information through various patient reported outcomes so that we can help them in various ways.” (34-year-old male in the occupational health setting with 10 years of experience) |
| 1. Patients are well informed about all aspects of their condition and care (e.g., clinical status, progress, and prognosis). | 0, 0% | “Patient centered health care means that they as the patient are the "center of attention" and understand what is going on with them, their condition, what goals are throughout the rehab process and why they may not be improving or experiencing a roadblock in their recovery. It is crucial that the patient or athlete are continually involved in the process and understand all decisions that surround the process.” (34-year-old male in the professional sports setting with 9 years of experience) |
| 1. Patients can access their medical records. | “Because we don't show patients their information in our EPIC system.” (30-year-old male in the military/law enforcement/government setting with 4 years of experience) | 0, 0% |
| 1. Athletic trainers support and educate patients on autonomy and self-care. | 0, 0% | “A lot of what we do is focused on autonomy.” (28-year-old female in the college/university setting with 7 years of experience) |
| 1. Open and effective communication between patients and athletic trainers occurs. | 0, 0% | “Communication is one of the most important tools humans have and to develop trust between a patient and athletic trainer that communication must be there.” (32-year-old female in the professional sports setting with 11 years of experience) |
| 1. Athletic trainers have the skills and knowledge to provide quality healthcare to varied patient populations. | 0, 0% | “I think athletic trainers undervalue their own skillset. We are very gifted, amazing clinicians that adapt on the fly. We are just used to giving our services for a very low price, which needs to stop, and this area needs to improve.” (32-year-old male in the military/law enforcement/government setting with 9 years of experience)  “Athletic trainers do not just cover fields for athletic events. We are generally the medical professional on site that could get called to the aid of a referee, parent, grandparent, or sibling of one of our athletes. Our medical knowledge might be centered around sports and returning athletes to play, but our general medical knowledge translates to all populations.” (29-year-old male in the professional sports setting with 7 years of experience) |
| 1. Athletic trainers acknowledge the role of a social support system in the care of the patient. | “Athletic Trainers do not recognize our role as a health care provider that has a responsibility to understand how injuries and illnesses effect patients, and how it can affect their role in society or in social circles.” (35-year-old male in the occupational health setting with 14 years of experience) | “The role of social support among patients and athletic trainers is extremely important. A large part of treatment relies on mental health and coping abilities, so if a patient does not feel connected or feels alone, that will affect their recovery.” (31-year-old female in the college/university setting with 10 years of experience) |
| 1. Athletic trainers connect the social support system that the patient relies upon with necessary resources in or near the healthcare facility. | “It is difficult to communicate with or engage with the patients social support system in this setting.” (27-year-old female in the occupational health setting with 5 years of experience) | 0, 0% |
| 1. Athletic trainers involve the patient’s social support system in decisions about the patient’s care. | “When we see patient's they often times do not have their social support system available in that moment and although we give the athletes the option of taking time to make a decision about treatment (specifically when it involves more invasive practices) they simply take the medical recommendation from us or other members of our staff. So, it is not that were preventing them from being involved, but they are not involved most times.” (25-year-old male in the professional sports setting with 1 year of experience)  “I picked it because I do not really think that athletic trainers really get the social support involved when determining treatment. They might mention to the patient how people around them could help but they do not bring that support in to discuss actually treatment.” (25-year-old female in the military/law enforcement/government setting with 4 years of experience) | 0, 0% |
